# An economic way to achieve all-weather CO_2_ reduction

**DOI:** 10.1093/nsr/nwad330

**Published:** 2023-12-27

**Authors:** Junwang Tang

**Affiliations:** Department of Chemical Engineering, University College London, UK

Utilising solar power to convert carbon dioxide (CO_2_) into hydrocarbon fuels presents an eco-friendly method to address dependence on fossil fuels and contribute to a net-zero CO_2_ emission goal [1,2]. Given that the lifespan of photogenerated electrons typically ranges from sub-picoseconds to a few seconds [3], the photocatalytic process will cease rapidly once the light source is turned off. The practical application of the CO_2_ conversion process is further hindered by its dependence on sunlight absorption, considering the variable nature of solar energy availability during nighttime, or under overcast or rainy conditions [4]. Furthermore, there is an inherent mismatch between the times when solar energy is accessible and when it is needed, which is affected by fluctuations in daylight duration and changes in weather patterns [5,6]. While integrating photovoltaic cells with batteries and CO_2_ electrolytic cells can theoretically address unstable solar power supply problems, the compounded energy losses at each step often lead to low overall conversion efficiencies [4,7]. Therefore, developing a simple and stable photocatalytic system for CO_2_ conversion remains a challenge.

**Figure 1.**
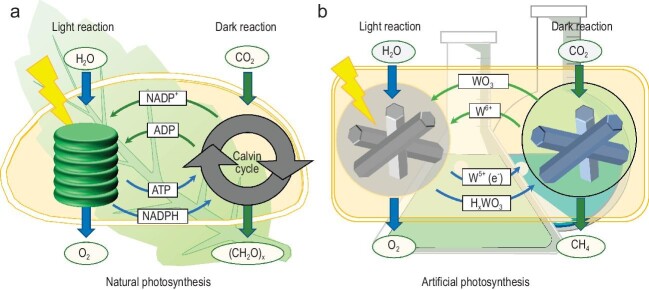
(a) The decoupled light-driven and dark reaction process of natural photosynthesis. (b) The decoupled light-driven and dark reaction process of artificial photosynthesis in this work. Adapted from ref. [8].

Writing in *National Science Review*, a joint team led by Professors Yu Huang, Junji Cao and Yujie Xiong overcome this critical issue by utilizing a single model material of Pt-loaded hexagonal tungsten trioxide (Pt/h-WO_3_) (Fig. 1) [8]. This catalyst demonstrates the ability to decouple light absorption process and CO_2_ reduction process by mimicking natural photosynthesis, and realises the sustainable CO_2_ conversion after the turn-off of light irradiation. The yield of CH_4_ using Pt/h-WO_3_ reaches 51.6 μmol/g in darkness following 10 minutes of simulated solar illumination. Characterisation has confirmed that the reduction of CO_2_ in the absence of light is initiated by the electrons and hydrogen atoms generated and stored in the catalyst during the preceding light irradiation. The unique properties of the h-WO_3_ support, including its capability to alternate between valence states (W^6+^/W^5+^) and its hexagonal channels, combined with the capacity of Pt to split water and transfer hydrogen atoms onto the h-WO_3_ surface, play a pivotal role in the successful decoupling of light and dark reactions in the process of solar-driven CO_2_ conversion. Furthermore, the authors constructed outdoor experimental equipments and conducted a 15-day continuous CO_2_ reduction experiment under natural light conditions. The data collected from the outdoor experiment show that the CO_2_ reduction process still works at night and during rainy periods, demonstrating successful all-weather CO_2_ conversion using a single material.

In summary, this work introduces a novel concept to achieve all-weather stable CO_2_ conversion by mimicking natural photosynthesis. While there still remains ample room for enhancing the conversion rate in the future, this work clearly establishes the viability of the approach to decouple light and dark reaction processes for sustainable solar-driven CO_2_ conversion. The implications of this work are profound, laying a foundation for advancements in eco-friendly energy solutions and bringing us closer to realizing a sustainable and resilient approach to CO_2_ conversion.

***Conflict of interest statement***. None declared.
